# Ocular perfusion pressure is not reduced in response to lower body negative pressure

**DOI:** 10.1038/s41526-024-00404-5

**Published:** 2024-06-08

**Authors:** Eric A. Hall, Richard S. Whittle, Ana Diaz-Artiles

**Affiliations:** 1 Department of Biomedical Engineering, Texas A&M University, College Station, TX USA; 2 School of Engineering Medicine (EnMed), Texas A&M University, Houston, TX USA; 3 Department of Mechanical and Aerospace Engineering, University of California Davis, Davis, CA USA; 4 Department of Aerospace Engineering, Texas A&M University, College Station, TX USA; 5 Department of Kinesiology and Sport Management, Texas A&M University, College Station, TX USA

**Keywords:** Outcomes research, Eye manifestations

## Abstract

Lower body negative pressure (LBNP) has been proposed as a countermeasure to mitigate the cephalad fluid shift occurring during spaceflight, which may be associated with the development of Spaceflight Associated Neuro-ocular Syndrome (SANS). This study quantifies the effect of LBNP on intraocular pressure (IOP), mean arterial pressure at eye level (MAP_eye_), and ocular perfusion pressure (OPP). Twenty-four subjects (12 male, 12 female) were subjected to graded LBNP in 0° supine and 15° head-down tilt (HDT) postures from 0 mmHg to –50 mmHg in 10 mmHg increments. IOP decreased significantly with LBNP pressure in 0° supine (by 0.7 ± 0.09 mmHg per 10 mmHg LBNP pressure, *p* < 0.001) and in 15° HDT (by 1.0 ± 0.095 mmHg per 10 mmHg of LBNP pressure, *p* < 0.001). MAP_eye_ significantly decreased by 0.9 ± 0.4 mmHg per 10 mmHg of LBNP pressure in 0° supine (*p* = 0.016) but did not significantly change with LBNP in 15° HDT (*p* = 0.895). OPP did not significantly change with LBNP in 0° supine (*p* = 0.539) but it significantly increased in 15° HDT at 1.0 ± 0.3 mmHg per 10 mmHg of LBNP pressure (*p* = 0.010). Sex did not have a significant effect on OPP, MAP_eye_, or IOP in any condition. In 15° HDT, the reduction in IOP during increasing negative pressure, combined with the relatively constant MAP_eye_, led to the increase in OPP. Furthermore, results suggest that LBNP, while effective in reducing IOP, is not effective in reducing OPP across all postures investigated.

## Introduction

Changes in the gravitational vector and posture induce fluid shifts within the body, which are hypothesized to contribute to a range of ocular manifestation during spaceflight. These fluid shifts result in altered pressures compared to upright postures^1,2^. The head is especially susceptible to fluid shifts during postural changes given its distance from the center of the body^3^. Cardiovascular compensatory mechanisms like the baroreflex enable the body to adapt well to postural changes in terrestrial conditions, specifically in maintaining proper blood perfusion to vital organs^4–6^. On the other hand, microgravity conditions are associated with cephalad fluid shifts and mild elevations in intraocular pressure and intracranial pressure (at least during short duration exposures) as well as jugular venous congestion^7–11^. It is hypothesized that these cephalad fluid shifts and subsequent pressure changes are contributing to a host of ocular conditions experienced by astronauts during long duration missions. These acquired symptoms include disk edema, choroidal folds, globe flattening, and hyperopic shifts in refractive error^12,13^. Collectively, this range of defects is known as Spaceflight Associated Neuro-ocular Syndrome (SANS)^14^. It has been hypothesized that SANS might be related to chronically, mildly elevated intracranial pressures (ICP) with respect to upright posture^12^. In addition, the absence of normal intracranial pressure cycles involving diurnal variations, as in terrestrial conditions, has also been hypothesized to have an important effect in the development of SANS^9,15^.

Lower Body Negative Pressure (LBNP) has been proposed as a countermeasure to mitigate SANS. LBNP is hypothesized to counteract the cephalad fluid shift occurring during spaceflight. LBNP mitigated the increase in optic nerve sheath diameter observed during head-down tilt (HDT) studies^16,17^ and partially mitigated increased choroidal engorgement during HDT bedrest studies when used overnight in eight-hour increments^18^. This finding should be interpreted with caution, however, as LBNP (‒25 mmHg) was not found to reduce optic nerve sheath diameter in spaceflight, pointing to the limitations of terrestrial analogs to spaceflight^19^. LBNP has also been used to study hypovolemia^20–24^, autonomic responses^23–25^, and cardiovascular compensatory mechanisms^22–24^. Additionally, LBNP in other long duration experiments has allowed for the study of neurohumoral responses to measure plasma norepinephrine, renin, and aldosterone levels^26^.

Prior work by Petersen et al. quantified gravitational influence on ocular pressures such as intraocular pressure (IOP), mean arterial pressure at eye level (MAP_eye_), and ocular perfusion pressure (OPP) during a graded tilt maneuver^27^. Ocular perfusion pressure (OPP), calculated as OPP = MAP_eye_ – IOP, is critical for ocular health as reductions in this metric are associated with the development and progression of glaucoma and reticular pseudodrusen associated choroidal thinning^28–30^. Additionally, possibly elevated OPP from hypertension and trauma incidents resulting in ocular hypotony have shown similar symptoms to SANS, including posterior globe flattening, a shift in refractive error, and optic disk edema^31–33^.

OPP changes can result from changes in MAP_eye_, IOP, or both. Postural changes resulting in slight head-down tilts, roughly analogous to microgravity environments, have been linked to increases in both IOP and MAP_eye_^27,34–36^. More recent studies have shown that the change in IOP between seated and supine postures is most similar to the change experienced during spaceflight^19,37–39^. IOP has previously been found to be dependent on episcleral venous pressures and as such, the systemic circulation^40^. Additionally, like MAP_eye_, IOP has been found to be higher in prone position with respect to supine position due to the presence of the additional hydrostatic column between the mid-coronal plane and the eye^27,41,42^.

The role of OPP in the development of SANS remains undetermined. However, we could speculate that the headward fluid shift experienced in spaceflight and the subsequent venous congestion^9,11^ result in a rise in the intravascular pressure within the vessels of the choroid, leading to increased capillary filtration of fluid, choroidal engorgement, and subsequent optic disc edema. Microgravity conditions could also lead to a rise in OPP, as arterial pressure is now equal to heart level due to the absence of a gravitationally dependent hydrostatic column. Elevated OPP could contribute to the extravasation of fluid because choroidal vessels could now be chronically exposed to slightly elevated pressures. Additionally, the prelaminar region of the optic nerve head lacks traditional blood–brain barrier characteristics, which could allow the filtration of fluid through vessel walls in this region^43^. Choroidal engorgement and increased total retinal thickness have been observed in astronauts, with optic disc edema from increased extravasation of fluid being previously proposed as a possible consequence of the cephalad fluid shift and venous congestion^13,38,44^.

While LBNP seems a promising countermeasure to mitigate the cephalad fluid shift and SANS, many open questions remain, including the characterization of the delicate balance between IOP and OPP, and their evolution across several LBNP levels. Thus, this study aims to quantify the effect of LBNP on intraocular pressure (IOP), mean arterial pressure at eye level (MAP_eye_), and ocular perfusion pressure (OPP) in different postures across a range of LBNP levels. This investigation provides insights into the effectiveness of LBNP in altering the pressures around the eye (IOP, MAP_eye_, OPP), which may have a role in the development of SANS^42^.

## Results

### LBNP tolerance

In the 0° supine position, two subjects showed presyncope symptoms at –40 mmHg (*n* = 2, both females) and 5 subjects showed presyncope symptoms at –50 mmHg (*n* = 5, 4 females, 1 male). In the 15° HDT position, one subject showed presyncope symptoms at –40 mmHg (*n* = 1, female), and another one showed presyncope symptoms at –50 mmHg (*n* = 1, female). Once the LBNP application was discontinued, subjects experienced no lasting symptoms. The data collected for these subjects before the interruption of protocol are included in the results.

### Dose-response curves

**Fig. 1:**
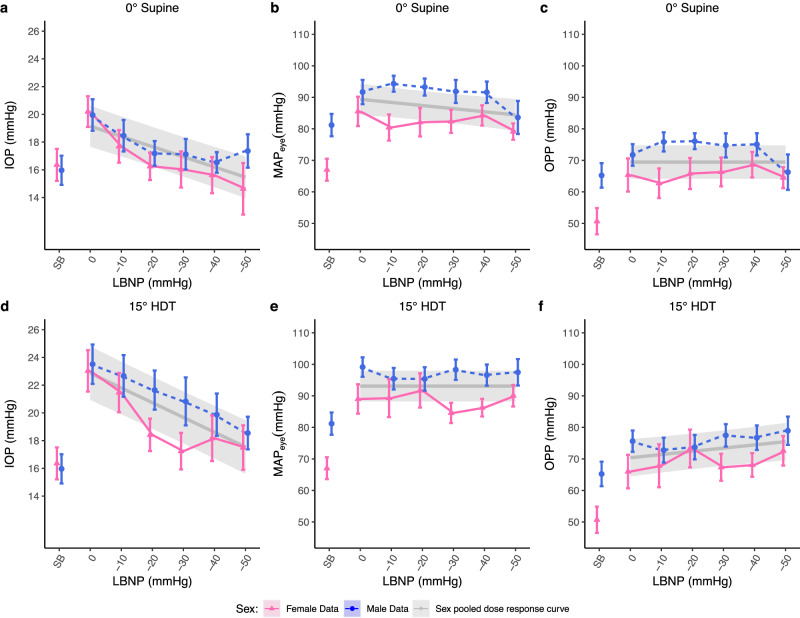
Intraocular pressure (IOP), mean arterial pressure at eye level (MAP_eye_), and ocular perfusion pressure (OPP) in 0° supine and 15° head down tilt (HDT) as a function of lower body negative pressure (LBNP) pressure on 12 male and 12 female subjects. Measurements were collected at seated baseline (SB), 0, ‒10, ‒20, ‒30, ‒40, and ‒50 mmHg. Experimental data for females (pink, solid) and male (blue, dashed) are shown as mean ± SE. Estimated dose-response curves fitted via linear mixed models (LMM) are shown in gray (mean ± 95% CI, male and female data pooled together). **a** IOP (0° supine), (**b**) MAP_eye_ (0° supine), (**c**) OPP (0° supine), (**d**) IOP (15° HDT), (**e**) MAP_eye_ (15° HDT), (**f**) OPP (15° HDT).

Side was found to have no significant effect on IOP values (*p* = 0.844). As such, side was removed from consideration and as a factor in the calculation of OPP values. Sex also did not have a significant effect on IOP, MAP_eye_, or OPP. Results for IOP, MAP_eye_, OPP, MAP, systolic blood pressure (SBP), and diastolic blood pressure (DBP) are shown in Table 1. Table 2 reports the results of linear mixed-effects model (LMM) analysis and Fig. 1 depicts the dose-response curves of IOP, MAP_eye_, and OPP as a function of LBNP pressure.

**Table 1:** Intraocular pressure (IOP), mean arterial pressure at eye level (MAP_eye_), ocular perfusion pressure (OPP), mean arterial pressure (MAP), systolic blood pressure (SBP), and diastolic blood pressure (DBP) during the experimental interventions (seated baseline, graded LBNP from 0 mmHg to –50 mmHg in 10 mmHg increments in 0° supine and 15° head-down tilt (HDT) postures)

Condition		Sex	IOP (mmHg)	SE	MAPeye (mmHg)	SE	OPP (mmHg)	SE	MAP (mmHg)	SE	SBP	SE	DBP	SE
											(mmHg)		(mmHg)	
Seated baseline	–	Females	16.36	1.15	67.04	3.47	50.69	4.18	89.22	3.37	117.26	4.3	77.41	3.48
		(*n* = 12)												
		Males	15.97	1.05	81.19	3.54	65.23	3.89	106.79	3.58	135.79	4.02	87.58	2.57
		(*n* = 12)												
0° Supine	0 mmHg	Females	20.2	1.11	85.55	4.66	65.35	5.25	91.12	4.6	118.25	2.95	76.69	2.53
		(*n* = 12)												
		Males	19.95	1.14	91.68	3.85	71.73	3.43	98.59	3.89	134.68	6.03	79.14	3.82
		(*n* = 12)												
	-10 mmHg	Females	17.69	1.18	80.43	4.15	62.74	4.71	86	4.11	120.85	4.03	77.22	1.39
		(*n* = 12)												
		Males	18.46	1.14	94.31	2.53	75.85	3.03	101.22	2.62	131.42	2.78	80.18	3.09
		(*n* = 12)												
	-20 mmHg	Females	16.25	0.99	82.09	4.52	65.84	4.94	87.66	4.48	123.41	2.65	76.36	1.99
		(*n* = 12)												
		Males	17.18	0.89	93.27	2.69	76.08	2.53	100.18	2.73	131.72	4.23	82.75	2.35
		(*n* = 12)												
	-30 mmHg	Females	16.03	1.3	82.34	3.65	66.32	4.57	87.91	3.62	115.49	3.4	74.15	2.33
		(*n* = 12)												
		Males	17.13	1.1	91.88	3.66	74.75	3.83	98.79	3.66	128.58	3.81	81.37	3.05
		(*n* = 12)												
	-40 mmHg	Females	15.62	1.31	84.25	3.22	68.64	4.05	89.96	3.22	115.22	2.7	74.37	2.02
		(*n* = 10)												
		Males	16.53	0.74	91.61	3.41	75.08	3.52	98.52	3.4	127.35	3.3	81.14	3.59
		(*n* = 12)												
	-50 mmHg	Females	14.63	1.86	79.14	2.58	64.51	3.32	84.88	2.58	115.8	2.85	78.38	3.26
		(*n* = 6)												
		Males	17.36	1.2	83.62	5.23	66.26	5.62	90.59	5.21	118.03	5.85	76.97	5.06
		(*n* = 11)												
15° head down tilt	0 mmHg	Females	23.03	1.49	89.01	4.67	65.98	5.31	88.66	4.64	113.51	2.6	74.91	2.28
		(*n* = 12)												
		Males	23.51	1.42	99.14	3.09	75.63	3.39	99.2	3.15	129.63	4.59	80.66	2.8
		(*n* = 12)												
	-10 mmHg	Females	21.46	1.42	89.29	6.05	67.83	6.83	88.99	6.03	106.91	2.12	72.22	0.97
		(*n* = 12)												
		Males	22.67	1.5	95.45	3.42	72.79	3.93	95.51	3.51	127.12	3.38	76.99	3.57
		(*n* = 12)												
	-20 mmHg	Females	18.42	1.17	91.72	5.43	73.31	6	91.37	5.4	124.02	4.15	77.4	2.16
		(*n* = 12)												
		Males	21.65	1.41	95.38	3.71	73.73	3.88	95.43	3.8	126.89	4.54	78.77	3.46
		(*n* = 12)												
	-30 mmHg	Females	17.24	1.32	84.59	3.2	67.34	4.27	84.23	3.22	123.22	3.15	79.25	3.52
		(*n* = 12)												
		Males	20.83	1.73	98.3	3.24	77.48	3.56	98.36	3.33	126.35	2.8	79.84	3.64
		(*n* = 12)												
	-40 mmHg	Females	18.17	1.65	86.26	2.81	68.09	3.75	85.96	2.81	119.68	4.76	79.25	4.56
		(*n* = 11)												
		Males	19.87	1.52	96.59	3.35	76.72	3.89	96.65	3.41	123.4	3.45	80.23	3.07
		(*n* = 12)												
	-50 mmHg	Females	17.5	1.6	90.07	3.42	72.57	4.67	89.73	3.39	116.21	3.24	73.01	2.53
		(*n* = 10)												
		Males	18.55	1.18	97.48	4.19	78.94	4.49	97.54	4.27	124.15	4.56	81.44	4.16
		(*n* = 12)												

Data are presented as mean ± SE with the number of subjects listed in each condition.

**Table 2:** Estimated model coefficients for the gravitational dose-response curves shown in Fig. 1 generated by linear mixed models (LMM)

			0° Supine			15° Head down tilt		
Metric	Model^a^	Units	*β*_*0*_ Intercept	*β*_*1*_ Pressure	Std dev of random effect^b^	*β*_*0*_ Intercept	*β*_*1*_ Pressure	Std dev of random effect^b^
IOP	LMM	mmHg	19.118 ± 0.75	−0.734 ± 0.095	3.413	22.836 ± 0.98	−1.064 ± 0.10	4.585
MAP_eye_	LMM	mmHg	89.146 ± 2.63	−0.992 ± 0.41	10.818	93.095 ± 2.49	–	11.74
OPP	LMM	mmHg	69.447 ± 2.70	–	12.82	70.367 ± 3.01	1.02 ± 0.39	13.62

Estimated coefficients are presented as a mean ± SE. Only significant terms are included in the models.

*IOP* intraocular pressure, *MAP*_eye_ mean arterial pressure at eye level, *OPP* ocular perfusion pressure

^a^All models use a linear predictor of the form: ${n}_{{ij}}={\beta }_{0}+{\beta }_{1}({{Pressure}}_{j})+{\gamma }_{i}\,+{\varepsilon }_{{ij}}$ for subjects *i (i* = 1:24) and LBNP pressure *j (j* = 0:5, 0 to –50 mmHg).

^b^Standard Deviation, $\sigma$, of random intercept, $\gamma$, for subject $i$. ${\gamma }_{i} \sim \left({0,\sigma }^{2}\right)$. Units for $\sigma$ are the same as the estimated coefficients.

### Intraocular pressure

IOP decreased significantly from 19.1 ± 0.75 mmHg at 0 mmHg to 15.4 ± 0.76 mmHg at –50 mmHg (i.e., 0.7 ± 0.090 mmHg per 10 mmHg of LBNP) in 0° supine (*p* < 0.001). IOP also decreased from 22.8 ± 0.9 mmHg at 0 mmHg to 17.5 ± 0.9 mmHg at –50 mmHg (i.e., 1.0 ± 0.095 mmHg per 10 mmHg of LBNP) in 15° HDT (*p* < 0.001).

### Mean arterial pressure at eye level

MAP_eye_ significantly decreased from 89.1 ± 2.6 mmHg at 0 mmHg to 84.2 ± 2.7 mmHg at –50 mmHg (i.e., 0.9 ± 0.4 mmHg per 10 mmHg of LBNP) in 0° supine (*p* = 0.016). However, in 15° HDT, MAP_eye_ did not significantly change across the negative pressures considered (*p* = 0.895).

### Ocular perfusion pressure

OPP did not change significantly in 0° supine between 0 mmHg and –50 mmHg (*p* = 0.539). In contrast, in 15° HDT, OPP was found to increase significantly, from 70.5 ± 3.1 mmHg at 0 mmHg to 75.5 ± 3.1 mmHg at –50 mmHg (i.e., 1.0 ± 0.3 mmHg per 10 mmHg of LBNP) (*p* = 0.010).

## Discussion

The effect of LBNP on IOP, MAP_eye_, and OPP was assessed over a range of graded LBNP pressures in both 0° supine and 15° HDT positions, and LBNP dose-response curves were generated for each of these parameters.

In 15° HDT, the reduction in IOP during increasingly negative LBNP pressure levels, combined with the relatively constant MAP_eye_, led to the increase in OPP. As determined in this study, OPP values are heavily reliant on MAP_eye_ values, which tend to have a disproportionate effect on OPP compared to the lower IOP pressures. In the 0° supine posture, both MAP_eye_ and IOP values decreased with more negative LBNP pressures, which resulted in the observed non-significant change in OPP.

It should be noted that while observed sex differences in MAP_eye_ were non-significant, there were significant sex differences in mean arterial pressure (MAP) in the study. Thus, there was a significant difference in the average MAP across all LBNP levels and postures. Males maintained an average MAP of 98.31 ± 12.5 mmHg (SBP: 128.15 ± 1.18 mmHg; DBP: 80.57 ± 0.94 mmHg) while females maintained an average MAP of 88.23 ± 13.7 mmHg (SBP: 117.87 ± 0.99; DBP: 76.19 ± 0.74 mmHg) (*p* < 0.001, Welch’s *t*-test, two-tailed, *α* = 0.05).

Six of the twelve females did not complete the protocol in either 0° supine or 15° HDT conditions due to discomfort or pre-syncope, and this typically occurred after the –40 or –50 mmHg pressure level. In contrast, only one male subject was unable to complete the full protocol (at 0° supine and –40 mmHg) due to presyncope. Women have previously been found to respond to LBNP differently than men due to lower physiological reserve to maintain arterial pressures^45^, reduced sympathetic responses to orthostatic stresses^46^, and greater pooling of blood in the pelvic region^47,48^. The etiology behind these sex disparities is still not fully understood and it warrants additional research prior to long duration space missions.

The exact mechanism behind SANS is still poorly understood. It has been hypothesized that SANS may be related to (mildly and permanently) elevated intracranial pressure (ICP) (compared to the upright posture)^12^. However, parabolic flight experiments utilizing subjects with Ommaya reservoirs have not shown an increase in direct ICP measurements upon acute exposure to microgravity conditions compared to supine postures^9^. Additionally, hypertension and terrestrial trauma incidents resulting in ocular hypotony have shown similar symptoms to SANS, including posterior globe flattening, a shift in refractive error, and optic disk edema^31–33^.

It should also be noted that OPP is significantly lower in seated, upright positions compared to both 0° supine and 15° HDT. Normal daily routines in 1 g conditions, which include the recommended 7–9 hours of sleep, typically consist in spending the majority of the time (up to 17 hours per day) in an upright position^49^. The transition from supine to upright posture introduces an approximately 13-16 mmHg decrease in OPP as shown in prior work^27^ and in this study, but may be as high as 20-30 mmHg^29^ (beyond the observed changes induced by the supine posture in this study). As was seen in case studies of ocular hypotony, a reduction of IOP, and possibly an increase in OPP, of ~12 mmHg in one eye was sufficient to cause the characteristic optic disk edema and posterior globe flattening typically present in SANS two months after the initial injury to the patient^31^. However, some consideration should be given as to whether elevated OPP or ocular hypotony were the cause of the findings similar to SANS. Petersen and colleagues noted the similarity between symptoms in cases where OPP was elevated through hypotony and cases of SANS, where there is increased IOP^27^. However, further research is warranted to elucidate the differences and similarities between these two mechanisms.

Perhaps most intriguing is the disparity between cases of elevated intracranial pressure on Earth and the symptoms that astronauts experience in space. A posterior displacement of the Bruch’s membrane opening during spaceflight has been observed in multiple studies, with 48% of astronauts in one study experiencing a posterior Bruch’s membrane opening displacement^38,50^. This result contrasts to terrestrial idiopathic intracranial-hypertension (IIH) patients, 80% of whom experienced anterior Bruch’s membrane displacement^50^. As was previously proposed, elevated OPP may contribute to enhanced extravasation of fluid into the interstitial space of the prelaminar optic nerve head, displacing the Bruch’s membrane in directions not normally seen in patients with elevated translaminar pressure gradients like IIH patients. It can therefore be hypothesized that chronically elevated OPP resulting from changes in hydrostatic forces may contribute to the optic disk edema observed in SANS.

As for LBNP being a suitable countermeasure to the development of SANS during spaceflight, further research is still warranted. LBNP has been shown to induce a shift in fluid volumes towards the lower body^51^, effectively reducing central venous pressure^52^. LBNP has also been shown to influence cerebrospinal fluid pressures surrounding the eye^17^ and to reduce choroidal engorgement^16^. In theory, LBNP would act as a viable countermeasure if it was able to reduce IOP and OPP back to the levels seen in seated upright postures. It has been previously established that LBNP induces reductions in central venous pressure^53,54^, central circulating blood volume^20,21,24^, and choroidal engorgement^18^. In this study, we observe reductions in IOP in both 15° HDT and 0° supine postures, as ocular humors are better able to flow into the venous circulation under elevated LBNP pressure conditions due to these established effects of LBNP. This enhanced outflow contributes to the observed pressure imbalance where IOP decreases while MAP_eye_ hypothetically remains elevated, which would lead to elevated OPP. Elevated OPP has previously been linked to symptoms similar to SANS such as optic disc edema, globe flattening, and a shift in refractive error^31–33^. Should elevated OPP be definitively linked to the optic disk edema observed in SANS, the use of LBNP could theoretically not be an effective countermeasure to this syndrome. Future work should seek to better understand the relationship between OPP and SANS, and the impact of LBNP on these ocular responses as part of countermeasure development.

These results should be extrapolated to microgravity conditions with caution. Physiological responses to LBNP on the ground (Earth) might differ to the physiological responses to LBNP in true microgravity conditions (or true altered-gravity conditions). In addition, our experimental conditions included 0° supine and 15° HDT, which might not exactly represent the headward fluid shift occurring in microgravity conditions. Previous work demonstrated that IOP measurements in 6° HDT were not significantly different from 0° supine^55^. Thus, we chose to use 15° HDT as an experimental condition (in addition to 0° supine) to induce a larger fluid shift to better quantify and understand the relationship between the amount of headward fluid shift and pressures around the eye; and this, at different levels of LBNP. While 15° HDT is not the traditional tilt angle to simulate microgravity conditions on Earth, prior studies have utilized 15° HDT in similar research, including post-spaceflight investigations^19,35^. However, the 0° supine IOP values obtained during this study most closely match the values obtained during spaceflight studies^39^.

Mean arterial pressure values collected in the seated baseline as well as normal pressure conditions in both 0° supine and 15° HDT during testing were high in males (all three conditions having MAP > 96 mmHg). Given the repeated calibration of the continuous blood pressure monitoring system with brachial cuff measurements and hypertension being an exclusionary criterion in the study, we surmise that the normotensive subjects were possibly excited or apprehensive about the impending pressure changes they were going to be subjected to when these measurements were taken.

MAP_eye_ calculations utilizing fluid columns represent an approximation of the OPP measurement. This study did not have access to direct measurements of arterial pressure and as such, the presented MAP_eye_ values may not be completely indicative of actual pressures within the body. Factors such as cerebral autoregulation and peripheral vasoconstriction, which may alter blood pressures collected at the fingers^26,56^, especially under orthostatic challenges like tilt and LBNP, exert some influence on MAP_eye_, but were not factored into the calculation of MAP_eye_. Additionally, LBNP pressures were not applied in a randomized order during the experimental sessions, which becomes another possible limitation of the study.

Furthermore, Buckey and colleagues found that body weight was strongly and positively correlated with changes in IOP values and potentially with the risk of developing SANS^57^. Reductions in weight, such as in terrestrial weight loss surgeries, were found to lead to reductions in IOP^57^. Buckey further hypothesized that this reduction also occur in space where microgravity conditions reduce the effective weight of an astronaut^58^. Reductions in weight are hypothesized to reduce IOP via a reduction in central venous pressure, an occurrence easily replicated in LBNP^52,58^. However, a more recent spaceflight study did not find any correlation between body weight and the development of optic dick edema, which is regularly associated with SANS, further implicating the multifactorial nature of SANS development and progression^59^. Concern over the accuracy of terrestrial LBNP experiments should be considered, as the combination of reduced central venous pressure while still experiencing G_x_ (front-to-back) gravity conditions may produce results not necessarily comparable to physiological responses during spaceflight conditions. Thus, future studies should investigate LBNP responses in true microgravity conditions.

In summary, LBNP reduces IOP in both 0° supine and 15° HDT but it also results in increased OPP in 15° HDT and no significant OPP change in 0° supine postures. Thus, during headward fluid shift (i.e., 15° HDT), the significant reduction of IOP due to negative pressures, combined with the relatively constant MAP_eye_, led to a significant increase in OPP with LBNP. While these changes in OPP are relatively small (and not significant in 0° supine), results suggest that LBNP is not effective in reducing OPP in 0° supine or 15° HDT.

## Methods

### Subjects and Study Approval

Twenty-four subjects (12 M/12 F, means ± SD: age 27.96 ± 2.88 yrs.; weight 74.47 ± 20.41 kg; height 169.58 ± 11.97 cm) participated in the study. All subjects provided written and oral consent prior to participating in the experiment. None of them took any medication (prescription or over the counter) at the time of the study, and they were instructed to avoid strenuous exercise and caffeine 12 hours before any of the experimental sessions. The subjects self-reported no history of chronic cardiovascular or ocular conditions (e.g., glaucoma) and no ocular surgery (including keratomileusis/keratectomy) within the previous 12 months. Subjects were recruited from a call for volunteers across the Texas A&M University System to participate in the study. From an initial pool of 100+ volunteers, the age range of selected subjects was limited as much as possible to avoid confounding factors related to changes in the cardiovascular system with age. The initially selected subjects were then screened for eligibility as per the exclusion criteria presented above. The protocol was discontinued immediately if subjects experienced any discomfort or symptoms of presyncope. The protocol was approved by the Institutional Review Board at Texas A&M University (TAMU) with IRB number IRB2020-0724F and was in accordance with the declaration of Helsinki.

### Procedure

**Fig. 2:**
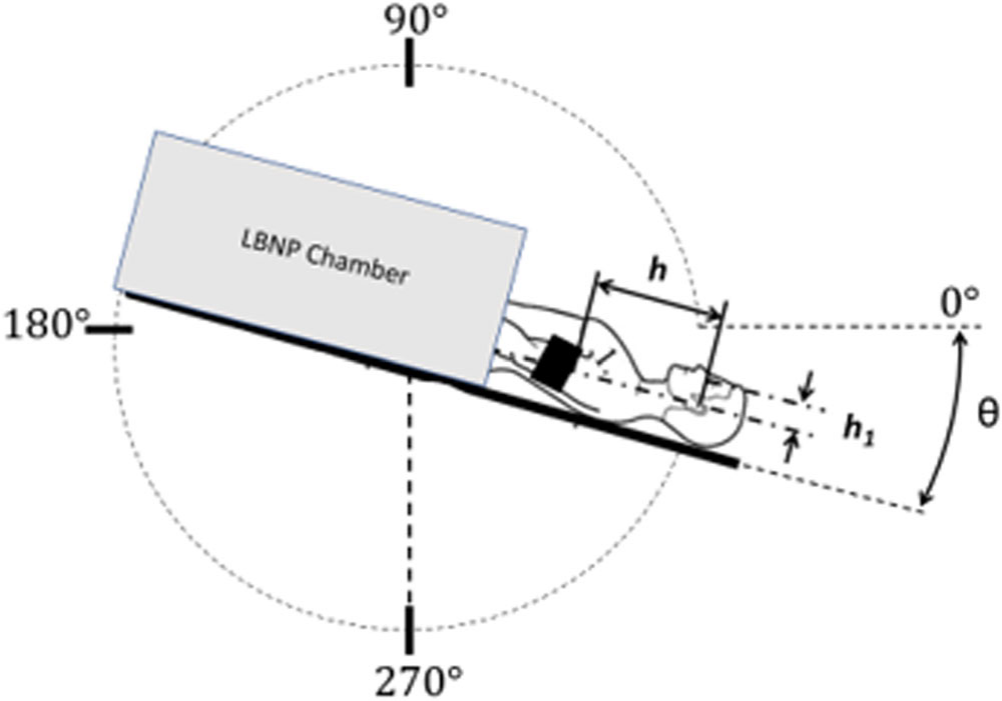
Experimental setup during the experimental sessions. Subject is placed in a lower body negative pressure (LBNP) chamber and subjected to graded negative pressure, from 0 mmHg to ‒50 mmHg, in 10 mmHg increments. The protocol is conducted twice: 0° supine (*ϴ* = 0°) and at 15° head down tilt (HDT) (*ϴ* = 15°) in a counterbalanced order. *h* represents the distance from heart to eye level while *h*_*1*_ represents the distance from the globe of the eye to the mid-coronal plane.

Subjects were placed in an LBNP chamber (Technavance; Austin, TX) and, after an initial five-minute period of rest, they were subjected to graded LBNP from 0 mmHg to –50 mmHg, in 10 mmHg increments. This procedure was repeated twice on separate days (order counterbalanced across subjects): once at 0° supine and once at 15° head-down tilt (HDT). These postures allowed for the collection of a range of applicable data based on their previous utilization in SANS-related research^19,37–39,60^. Figure 2 shows a schematic of the experimental configuration. A five-minute acclimatization period was provided after each change in LBNP pressure to ensure steady state conditions had been reached and the effect of time was minimized. After the five-minute acclimatization period, blood pressure and bilateral IOP measurements were collected. Prior work has demonstrated that short duration LBNP (3–5 minutes) induces predominantly autonomic nervous reflexes, which were observed in the larger study but are beyond the scope of this paper^26,56,61^. Given that autonomic nervous reflexes predominately control body responses to LBNP during the time frame tested in this experiment and the widely used and repeatable nature of graded LBNP approaches in research, this technique was chosen for this experiment^26,56,61–66^. Mean arterial pressure at eye level (${{\rm{MAP}}}_{{\rm{eye}}}$) was calculated using Eq. (1):

1 $${{\rm{MAP}}}_{{\rm{eye}}}={\rm{MAP}}-{\rm{\rho }}{\rm{g}}h\sin \left({\rm{\theta }}\right)-{\rm{\rho }}{\rm{g}}{h}_{1}\cos ({\rm{\theta }})$$

where MAP denotes mean arterial pressure, rho (*ρ*) represents the density of blood (*ρ* = 1060 kg m^–3^*)*, *g* denotes acceleration due to gravity (*g* = 9.81 m s^–2^*)*, *h* represents the height from heart to eye level (cm), *h*_*1*_ represents the distance from the mid coronal plane to the globe of the eye (cm), and theta (*ϴ*) denotes the angle of tilt with respect to the 0° supine position (degrees)^27,67^.

Mean arterial pressure (MAP) was measured continuously via plethysmography using a cuff around the third finger on the subjects’ left hand (Finapres NOVA, Finapres Medical Systems B.V.; Enschede, Netherlands) and corrected to heart level using the Finapres height sensor, which was attached to the subjects at the fifth intercostal space on the mid-coronal plane. Brachial blood pressure calibrations using the Finapres NOVA arm cuff were performed after each LBNP pressure transition and the respective five-minute acclimatization period. MAP_eye_ was then calculated by accounting for the hydrostatic fluid column over the distance between heart and eye level (*h* in Fig. 2) and the midcoronal plane and anterior portion of the eye globe (*h*_*1*_ in Fig. 2). Both distances were measured on each subject before the experimental sessions.

Intraocular pressure (IOP) was measured in both eyes via the rebound technique using an IC200 rebound tonometer (iCare Finland Oy; Vantaa, Finland). Each IOP value was calculated as the average of the central four of six measures. This procedure was repeated at each LBNP pressure level in the two postures investigated (0° supine and 15° HDT).

### Statistical analysis

Data and statistical analysis were performed in R version 4.1.0 (R foundation for statistical computing, Vienna, Austria). Results are presented as mean ± SE. Dose-response models quantifying the effects of pressure and sex on IOP, MAP_eye_, and OPP at the different postures were constructed over the range of LBNP pressures considered using linear mixed-effects models (LMM). Diagnostics plots for all models were examined visually and statistically to confirm normality and homoscedasticity of residuals. In all models (i.e., IOP, MAP_eye_, and OPP), LBNP pressure and sex (male, female) were used as fixed effects, whilst the subjects were included as a random effect. In addition, side (left vs right) was also included as a fixed effect for IOP and OPP. Tilt postures (i.e., 0° supine and 15° HDT) were modeled separately given the already established significant effects of tilt angle on the metrics studied here^27^. The general equation of the models fitted to each parameter is shown in Eq. (2):

2 $${n}_{{ijkl}}={\beta }_{0}+{\beta }_{1}({{\rm{Pressure}}}_{{\rm{j}}})+{\beta }_{2}({{\rm{Sex}}}_{{\rm{k}}}){+\beta }_{3}({{\rm{Side}}}_{{\rm{l}}})+{\gamma }_{i}\,+{\varepsilon }_{{ij}}$$

where the linear predictor ${n}_{{ijkl}}$ for each subject *i (i* = 1:24) of sex *k* (*k* = 1:2, male and female) in each respective tilt posture is described by the LBNP pressure *j* (*j* = 0:5, 0 to –50 mmHg) and side *l* (*l* = 1:2, left and right) where appropriate. The terms *β* represent the fixed effect coefficients, where ${\beta }_{0}$ represents the intercept, and *γ*_*i*_ represents the subject specific random intercept. ${\varepsilon }_{{ij}}$ represents the residual error of the predictor. Where there was no significant effect of sex, the results from male and female subjects were combined to a single dose-response model. Similarly, where there was no effect of side, the results from the left and right sides were also combined. Dose-response curves are shown as mean and 95% confident band. The level of statistical significance was set to $\alpha =0.05$ (two-sided).

### Reporting summary

Further information on research design is available in the Nature Research Reporting Summary linked to this article.

### Supplementary information

Reporting Summary

## Data Availability

The data collected and used in this study are available on GitHub at https://github.com/BHP-Lab/LBNP-OPP/.

## Code availability

The code developed and used in this study is available upon request.
