# Supplementary material for: Ocular perfusion pressure is not reduced in response to lower body negative pressure
Source: NPJ Microgravity. 2024 Jun 8;10:67. doi: 10.1038/s41526-024-00404-5 (PMC11162494; doi:10.1038/s41526-024-00404-5)
Supplement: Supplementary file 1 — Reporting Summary [file 41526_2024_404_MOESM1_ESM.pdf]

nature portfolio

Corresponding author(s): Ana Diaz-Artiles

Last updated by author(s): May 4, 2024

# Reporting Summary

Nature Portfolio wishes to improve the reproducibility of the work that we publish. This form provides structure for consistency and transparency in reporting. For further information on Nature Portfolio policies, see our Editorial Policies and the Editorial Policy Checklist.

## Statistics

For all statistical analyses, confirm that the following items are present in the figure legend, table legend, main text, or Methods section.

| n/a | Confirmed | |
|---|---|---|
| ☐ | ☒ | The exact sample size (*n*) for each experimental group/condition, given as a discrete number and unit of measurement |
| ☐ | ☒ | A statement on whether measurements were taken from distinct samples or whether the same sample was measured repeatedly |
| ☐ | ☒ | The statistical test(s) used AND whether they are one- or two-sided<br>*Only common tests should be described solely by name; describe more complex techniques in the Methods section.* |
| ☐ | ☒ | A description of all covariates tested |
| ☐ | ☒ | A description of any assumptions or corrections, such as tests of normality and adjustment for multiple comparisons |
| ☐ | ☒ | A full description of the statistical parameters including central tendency (e.g. means) or other basic estimates (e.g. regression coefficient) AND variation (e.g. standard deviation) or associated estimates of uncertainty (e.g. confidence intervals) |
| ☐ | ☒ | For null hypothesis testing, the test statistic (e.g. *F*, *t*, *r*) with confidence intervals, effect sizes, degrees of freedom and *P* value noted<br>*Give P values as exact values whenever suitable.* |
| ☒ | ☐ | For Bayesian analysis, information on the choice of priors and Markov chain Monte Carlo settings |
| ☐ | ☒ | For hierarchical and complex designs, identification of the appropriate level for tests and full reporting of outcomes |
| ☐ | ☒ | Estimates of effect sizes (e.g. Cohen's *d*, Pearson's *r*), indicating how they were calculated |

*Our web collection on statistics for biologists contains articles on many of the points above.*

## Software and code

Policy information about availability of computer code

| | |
|---|---|
| Data collection | No software was used, except for the software embedded in the devices used for data collection, described in the manuscript (LBNP Technavance, Finapres NOVA, Icare IC200 rebound tonometer) |
| Data analysis | Data and statistical analysis were performed in R version 4.1.0 (R foundation for statistical computing, Vienna, Austria). |

For manuscripts utilizing custom algorithms or software that are central to the research but not yet described in published literature, software must be made available to editors and reviewers. We strongly encourage code deposition in a community repository (e.g. GitHub). See the Nature Portfolio guidelines for submitting code & software for further information.

## Data

Policy information about availability of data

All manuscripts must include a data availability statement. This statement should provide the following information, where applicable:

- Accession codes, unique identifiers, or web links for publicly available datasets
- A description of any restrictions on data availability
- For clinical datasets or third party data, please ensure that the statement adheres to our policy

The data collected and used in this study are available on GitHub at https://github.com/BHP-Lab/LBNP-OPP/

## Research involving human participants, their data, or biological material

Policy information about studies with human participants or human data. See also policy information about sex, gender (identity/presentation), and sexual orientation and race, ethnicity and racism.

| | |
|---|---|
| Reporting on sex and gender | Both male and female sex subjects were recruited for this study. The total number of subjects was 12 male and 12 female subjects. All subjects were informed that de-identified aggregate data separated by sex would be reported in the published outcomes of the study. |
| Reporting on race, ethnicity, or other socially relevant groupings | No analysis based on race, ethnicity, or socially relevant groupings was performed in this study. All data were separated purely on the basis of sex given the well established differences in physiology and anatomy these two groups possess. |
| Population characteristics | Twenty-four subjects (12 male/12 female, means ± SD: age 27.96 ± 2.88 yrs.; weight 74.47 ± 20.41 kg; height 169.58 ± 11.97 cm) were included in this study. |
| Recruitment | Subjects were recruited from a call for volunteers across the Texas A&M University System to participate in the study. From an initial pool of 100+ volunteers, the age range of selected subjects was limited as much as possible to avoid confounding factors related to changes in the cardiovascular system with age. The initially selected subjects were then screened for eligibility as per the inclusion/exclusion criteria. |
| Ethics oversight | The protocol was approved by the Institutional Review Board at Texas A&M University (TAMU) with IRB number IRB2020-0724F and was in accordance with the declaration of Helsinki. |

Note that full information on the approval of the study protocol must also be provided in the manuscript.

# Field-specific reporting

Please select the one below that is the best fit for your research. If you are not sure, read the appropriate sections before making your selection.

☒ Life sciences ☐ Behavioural & social sciences ☐ Ecological, evolutionary & environmental sciences

For a reference copy of the document with all sections, see nature.com/documents/nr-reporting-summary-flat.pdf

# Life sciences study design

All studies must disclose on these points even when the disclosure is negative.

| | |
|---|---|
| Sample size | A power analysis was conducted on preliminary data from a previous linked experiment (Whittle et al., J Am Heart Assoc 2022), where it was determined that 24 subjects (12 male, 12 female) were sufficient to develop a dose-response curve with sufficient power. The same subjects were used in this experiment, which is part of a much larger study investigating cardiovascular dose-response in altered-gravity environments. In general, the effect sizes from LBNP were larger than those from the tilt study in Whittle 2022, thus it was determined that no further subjects needed to be recruited. |
| Data exclusions | Two subjects displayed symptoms of presyncope at –40 mmHg, and a further five subjects at –50 mmHg. Data collection for those subjects was terminated at that point, however all data collected up to termination are included in the publication. |
| Replication | This manuscript presents the first dose-response models constructed from experimental data. The totality of the data are used to construct the models. We present full statistical models to enable future studies to replicate the findings, however no replication was included in this study. |
| Randomization | All subjects were exposed to both supine and head down tilt postures on different days along with the same data collection protocols across all experiments. Subjects were randomized in which order they were exposed to one posture or another (i.e., some supine first and others head down tilt first). In all postures and days, the same amount of lower body negative pressure was used in a stepwise manner beginning at 0 mmHg and finishing at 50 mmHg utilizing 5 minute acclimatization periods followed by 10 minute sample collection periods. |
| Blinding | Blinding was not possible during this experiment due to the shortage of people capable of collecting data. In all sessions, the primary researchers were present and collecting subject data with the assistance of several rotating counterparts who participated based on their availability. The lack of blinding should not affect the outcomes of this study as the researchers were careful not to disclose what the expected results of the testing were to subjects at any point during the testing and subjects were encouraged to halt testing at any point if they felt uncomfortable. |

# Reporting for specific materials, systems and methods

We require information from authors about some types of materials, experimental systems and methods used in many studies. Here, indicate whether each material, system or method listed is relevant to your study. If you are not sure if a list item applies to your research, read the appropriate section before selecting a response.

## Materials & experimental systems

| n/a | Involved in the study |
| --- | --- |
| ☒ | ☐ Antibodies |
| ☒ | ☐ Eukaryotic cell lines |
| ☒ | ☐ Palaeontology and archaeology |
| ☒ | ☐ Animals and other organisms |
| ☒ | ☐ Clinical data |
| ☒ | ☐ Dual use research of concern |
| ☒ | ☐ Plants |

## Methods

| n/a | Involved in the study |
| --- | --- |
| ☒ | ☐ ChIP-seq |
| ☒ | ☐ Flow cytometry |
| ☒ | ☐ MRI-based neuroimaging |

# Plants

| | |
| --- | --- |
| Seed stocks | N/A |
| Novel plant genotypes | N/A |
| Authentication | N/A |
